# The Neurological Exam of a Comatose Patient: An Essential Practical Guide

**DOI:** 10.21315/mjms2020.27.5.11

**Published:** 2020-10-27

**Authors:** Zaitun Zakaria, Mohamad Muhaimin Abdullah, Sanihah Abdul Halim, Abdul Rahman Izaini Ghani, Zamzuri Idris, Jafri Malin Abdullah

**Affiliations:** 1 Department of Neurosciences, School of Medical Sciences, Universiti Sains Malaysia, Kubang Kerian, Kelantan, Malaysia; 2 Department of Neurosciences, Hospital Universiti Sains Malaysia, Universiti Sains Malaysia, Kubang Kerian, Kelantan, Malaysia; 3 Brain and Behaviour Cluster, School of Medical Sciences, Universiti Sains Malaysia, Kubang Kerian, Kelantan, Malaysia; 4 Department of Neurosurgery, Hospital Sultanah Bahiyah, Alor Setar, Kedah, Malaysia; 5 Neurology Unit, Department of Internal Medicine, School of Medical Sciences, Universiti Sains Malaysia, Kubang Kerian, Kelantan, Malaysia

**Keywords:** comatose, consciousness, meningism, cranial nerves, motor assessment

## Abstract

A thorough examination of a comatose patient is essential given the spectrum of clinical diagnoses. The most immediate threat to patients is airway, breathing and circulation. All attending physician should employ a structured and focused approach in dealing with a comatose patient. It is important to recognise the urgent steps needed at the time to prevent further deterioration, followed by the final diagnosis of patient’s neurologic status. Here we provide the essential practical guide to the neurological exam of a comatose patient that would assist to determine the aetiology, location and nature of the neurological lesion.

## Introduction

The evaluations of the comatose patients require a stepwise approach starting with a history, physical examination and laboratory evaluation. The causes of coma may be reversible when detected early. It therefore seems pertinent that once we confirmed an unobstructed airway, that the patient is breathing, and that there is normal circulatory function, a structured and focused examination must be undertaken (1). The examiner should determine where is the lesion responsible for coma, the nature and what is the urgent steps that are needed at the time to prevent further neurological damage. In the neurological exam of a comatose patient, the outline includes: i) general examination; ii) level of consciousness; iii) cranial nerves; and iv) motor assessment.

## General Examination

The examiner must have a systematic and thorough examination. The general examination starts with observing the stationary position of the patient on the bed and attitude of the limb. It should be documented if there is any spontaneous motor behaviour or semi-purposive movements of all four extremities, breathing pattern and oropharyngeal reflexes such as coughing, swallowing, hiccupping or yawning. Inspection for clues for trauma such as bleeding, scars, track marks and post-operative drainage catheters may indicate the site of injury. In intensive care environment, all connected intravenous infusion is checked for sedative agents and or vasopressors. This is important if there is a question as to whether a drug or intervention has an effect to patient’s conscious level. When the patient is on a mechanical ventilator, the settings give a clue if any spontaneous breaths are taken by the patient.

## Level of Consciousness

The current recommendation by the European Academy of Neurology (EAN) (2) is that the Full Outline of UnResponsiveness (FOUR) score (Table 1) (3) is to be used for a comatose patient in intensive care unit (ICU) setting, instead of the Glasgow coma scale (GCS) score (Table 2) (4). The examiner must document what the patient did in response to particular stimuli. The stimuli are either peripheral or central.

### Peripheral Painful Stimuli (as described by Teasdale and Jennett (4))

This technique is used to elicit an eye-opening response.

#### Interphalangeal joint pressure (IJP)

- Apply pressure with a pencil or pen to the lateral outer aspect of the proximal or distal interphalangeal joint for 10 s–15 s to elicit a response.
- A peripheral painful stimulus may elicit a spinal reflex, causing flexion of the tested limb; a spinal reflex is not an indication of intact brain function.

### Central Painful Stimuli (as described by Teasdale and Jennett (4))

This technique is used to elicit a motor response. It is done by stimulating a cranial nerve, thus avoiding the possibility of eliciting a spinal reflex.

#### Trapezius twist (cranial nerve XI)

- This technique uses the thumb and two fingers as pincer
- First, take hold of about two inches of the muscle located at the angle where the neck and shoulder meet
- Then, twist and gradually apply increasing pressure for 10 s–20 s to elicit a response. High level spinal cord injuries may interfere with assessment using the trapezius twist

#### Supraorbital pressure (cranial nerve V)

- First, place the flat of the thumb on the supra-orbital ridge (small notch below the inner part of eyebrow), while the other hand rests on the head of the patient
- Then, gradually apply an increasing pressure for 10 s–20 s to elicit a response. It is important to make sure that the patient does not have orbital fracture, facial fractures, or frontal craniotomies

#### Jaw margin pressure (cranial nerve V) (first described by Wijdicks (5))

- First, place the flat of the thumb on both condyles at the level of the temporomandibular joint
- Gradually apply an increasing pressure for 10 s–20 s to elicit a response

#### Sternal rub

- This technique is usually performed when the previously mentioned stimuli are unable to elicit a response
- A midline stimulus is applied using the knuckles of a closed fist onto the patient’s mid sternum (not the xiphoid process)
- The pressure is applied for a maximum of 30 s and caution should be taken in patients with previous cardiac surgery or signs of injury to the sternum

### Meningismus

In neurological condition that causes irritation to meninges, such as meningitis and subarachnoid haemorrhage, bedside diagnostic signs can be used for evaluation. The presence of meningeal irritation, however, is not pathognomonic for meningitis (6). The signs that can be elicited include:

#### Neck stiffness/nuchal rigidity (7)

- With the patient lying on his or her back and both legs fully extended at the knee, an attempt is made to flex the patient’s neck towards the chest
- Due to spasm of the extensor muscles of the neck, there will be resistance to passive flexion of the head (stiffness)
- The inability to either touch the chin to the chest or lift the head 8 cm off the bed when supine is used for definition
- The lateral neck movements are preserved (*versus* neck rigidity from musculoskeletal pain)

#### Kernig’s sign (8–11)

- With the patient lying on his or her back and both legs fully extended at the knee, a two-stage attempts is performed with flexing of lower limb simultaneously at the hip and knee, then extending the knee
- Positive test is when there is resistance to the test and or involuntary flexion of the opposite hip

#### Brudzinski’s sign (8, 12)

- With the patient lying on his or her back and both legs fully extended at the knee, keep one hand behind the patient’s head and the other on the chest in order to prevent the patient from rising
- An attempt is made to flex the patient’s neck towards the chest; a positive test is when the patient involuntarily flexes his or her hips and knees in an attempt to minimise the meningeal irritation

#### Lasègue’s sign (as published by Wartenberg (9), and Maranhão-Filho and Vincent (13))

- With the patient lying on his or her back and both legs fully extended at the knee, the examiner passively flexes each leg at the hip while keeping the knee joint extended
- Normally the straight leg can be lifted to an angle (with horizontal) of 65°–90° position
- Positive test is when the angle is reduced as well as pain and limitation of movements present earlier

The sign of meningism may present bilaterally in patients with meningeal irritation and unilaterally in patients with unilateral sciatica. Whenever Kernig’s sign is positive, Lasègue’s sign is generally also found to be positive and vice versa.

## Cranial Nerves

### Vision (Cranial Nerve II)

Assessment of vision includes voluntary eye movements, visual pursuit and blink-to-threat techniques.

#### Voluntary eye movements (2)

- The test is done by passively opening the eyes of the patient without stimulation to trigger the eye opening
- Then, look for spontaneous eye movement, or eye movement on command. If there was no response, then proceed for visual pursuit

#### Visual pursuit (2)

- The test is done using a bedside tool such as a mirror. Put the mirror in front of the patient’s face to evoke a response and moves it horizontally and vertically
- Observe for evidence of visual tracking

#### Blink-to-threat (14)

- The test is done by passively opening the eyes of the patient, then move your fingers rapidly towards the patient’s eyes to see if a blink occurs
- This test is done in all four direction

### Pupillary Assessment and Light Reflex (Cranial Nerves II, III)

#### Position

Upon lifting the patient’s eyelids, the examiner should notice the shape and position of the pupils, whether it is central, deviated or dysconjugate gaze. Examples of findings include:

- A frontal lobe lesion, commonly a stroke: the eyes deviation is on the same side of the lesion
- Todd’s paralysis following a seizure: the eyes deviate in the opposite direction
- A pontine lesion or thalamic haemorrhage: the eyes deviate in the opposite direction

#### Pupillary response

Pupillary light reflex (PLR) is simply the change in pupil size that occurs after a light stimulus. It provides information on the brainstem integrity in the comatose patients (15). A transient flash of light within 3 s–5 s will produce a decrease in pupil size. PLR is generated by smooth muscle and is unaffected by neuromuscular blocking drugs. However, the examiner has to ensure whether the patient is on sedative agent and the level of sedation, since the sympathetic contribution to pupil size may be absent due to anaesthetic-induced miosis (15, 16). It is also useful to check whether the patient is on any pupil dilator medication or has an underlying optic nerve injury, such as traumatic optic neuropathy.

It has previously been recommended to use the NeurOptics portable pupillometer for bedside assessment. The device measures the Neurological Pupil index (NPi) to determine each pupil assessment (15); the NPi is scored from 0 (nonreactive) to 5 (brisk) with values < 3 considered to be sluggish or abnormal. The pupillary response should include direct, indirect and rapid afferent pupillary defect (RAPD). Examples of the findings include:

- Right putaminal haemorrhage: eyes are deviated to the side of lesion. Pupils will be normal size and reactive
- Thalamic haemorrhage: eyes are looking down at the nose. Pupils will be small and nonreactive
- Pontine haemorrhage: eyes are in mid-position with no movement to doll’s eyes manoeuvre. Pupils will be pinpoint

### Ophthalmoscopic Exam (Cranial Nerve II)

The steps are as follows:

- Darken the room
- Approach the patient – use right hand for the patient’s right eye
- From a 15° lateral, look into the patient’s eye. Check for presence of red reflex
- Then, aim your view towards the nasal
- When you see blood vessels, follow nasally toward retina and optic disc
- Check for colour, contour and cupping
- Comment on vasculature and 4 quadrants

### The Eyelid-Release Test (Cranial Nerve VII) (17)

The steps are as follows:

- While standing at the head of the patient’s bed, elevates both lids and releases them simultaneously
- The lid of the hemiplegic side closes slowly because of the flaccidity of its orbicularis oculi muscle, whereas the lid of the normal side closes briskly because of tonus in its orbicularis oculi muscle

### Oculocephalic Manoeuvre (Doll’s Eye Test) (18, 19)

The steps are as follows:

- Ensure the C-spine is cleared
- The patient’s eyes are held open
- The head is briskly turned from side to side with the head held briefly at the end of each turn
- The degree of rotation must be more than 30° per second in order to generate enough velocity to overcome pursuit
- A positive response occurs when the eyes rotate to the opposite side to the direction of head rotation (cranial nerves III, VI, VIII)
- A similar result is seen when the head is flexed and extended — a positive result is the downward deviation of the eyes during extension, and upward deviation during flexion (cranial nerves III, IV, VIII)
- The eyes should gradually return to the mid-position in a smooth, conjugate movement if the brainstem is intact

### Corneal Reflex (Cranial Nerves V, VII) (20)

The steps are as follows:

- Stimulus is applied from the side while the patient is looking in the opposite direction
- Gently touching or stroking the cornea with a wisp of moistened cotton
- Positive results will elicit bilateral blinking of eyes
- Also observe for contralateral deviation of jaw (corneomandibular reflex) (21): tend to occur with firm stimulation of cornea

### Caloric Testing (first described by Barani (22))

The steps are as follows:

- The head is elevated to 30 degrees above horizontal so that the lateral semi-circular canal is vertical, so that stimulation will generate a maximal response
- Check that the tympanum is intact and that the external ear canal is clear

### Horizontal Oculovestibular Eye (Cranial Nerves III, VI, VIII)

- Introduce 50 mL of water into the external ear canal through a small catheter at a temperature of 7 °C above or below the normal body temperature (30 °C or 44 °C), flushed at a rate of 10 mL/min
- Allow 5 min between testing ears to allow re-equilibration of the oculovestibular system
- COWS: refers to direction of nystagmus (fast) component: cold opposite, warm same side
- Lateral gaze deviation from a pontine lesion cannot be overcome by stimulating oculocephalic or oculovestibular reflexes, whereas supranuclear (e.g. frontal lesions) can be overcome. Hence, they can be distinguished clinically

### Vertical Oculovestibular Eye (Cranial Nerves III, IV, VIII)

- Responses can be assessed by irrigating both ears simultaneously
- If the brainstem is intact, cold water causes the eyes to deviate downwards and warm water causes the eyes to deviate upwards

### Gag Reflex

- Inspect for asymmetry of uvula and palatal arch
- Touch one tonsillar pillar and then the other with a tongue blade
- The afferent arc of the gag reflex is primarily innervated by the glossopharyngeal nerve; the efferent arc is innervated by the vagus nerve

## Motor Assessment

The neurological assessment may be limited particularly when the patient is unable to follow steps command or respond to sensory examination (17, 23, 24). However, the examiner can still be able to perform certain manoeuvres and elicits signs. The motor assessment is divided into:

- Motor Tone
- Motor Responses
- Motor Reflexes
- Plantar Response

### Motor Tone

The grading can be done using the 1964 Ashworth scale (25), or the 1987 modified Ashworth scale by Bohannon and Smith (26) (Table 3). The assessment for tone is performed for upper limb, lower limb and neck.

#### Upper limb

- Hold the wrist with your left hand. Then, attempt to dorsiflexion and plantarflexion the wrist with the other hand
- Next, support the patient’s elbow. Then, hold patient’s hand as if shaking hands and attempt to rapidly supinate and pronate the arm
- Next, while still supporting the elbow, passively flex and extend the elbow
- This is followed by shoulder rotation
- Use the same technique on each arm

#### Lower limb

- Place your hand on the thigh and roll the whole legs. Observe the movement of the foot. If tone is normal, the range of movement of the foot is similar to the rotation of the leg
- Grasping each leg at the knee and gently lifting it from the bed. Observe the difference between each leg
- Place your left hand under the patient’s thigh. While holding the foot with the other hand, alternately extending and flexing the patient’s knee and ankle

#### Neck

- Gently grasping the head with two hands and moving it back and forth or up and down
- Another method is head dropping test (23) to test for rigidity: the examiner places one hand under the patient’s occiput and with the other hand briskly raises the head, and then allows it to drop. Normally, the head drops rapidly into the examiner’s protecting hand, but in patients with extrapyramidal rigidity, there is delayed and slow dropping of the head. When meningismus is present, there is resistance to pain on flexion of the neck

### Motor Responses

For movements with elimination of gravity, the head end of the bed is placed in 10° position. The arm and legs are put in a neutral position and are observed for weakness. The flaccid arm will be in extension with semiflexed fingers, while the leg will be in extension and externally rotated. Each muscle strength is tested and graded using the Medical Research Council (MRC) grading (27). Some examinations require a conscious patient, such as Holmes tremor (28) and Mingazzinni test (29, 30). Hence, in this scenario, we will show several tests that can be done to test muscle weakness.

#### Arm dropping test (Reeve and Bullen test) (31)

- With patient lying supine, put patient’s arm over the head and drops the weak arm
- The hemiplegic arm drops limply, whereas the normal arm glides or floats down
- This is also useful to test for psychogenic neurologic deficits, whereby in organic paresis the arm hits the face. However, in functional paresis a voluntary movement allows avoiding the face

#### Leg-dropping test (32)

- Crook the patient’s knees on your arm
- Extend one leg first and drop it, and do the same with the other
- Observe the difference as the flaccid hemiplegic leg drops more rapidly to strike the bed

#### Motor strength of lower limb (sign published by Vanpee et al. (33), adapted from Herman et al. (34))

- The ability of the patient to maintain a fixed posture of a lower limb for a few (3–5) seconds is regarded as power 3/5.
- E.g. the patient’s thigh is flexed with knee flexed at 90° and observe the ability of the patient to maintain the position.

#### Barré test (manoeuvre de la jambe)

- Known as ‘leg drift test’ (30, 35). In a prone position, the patient’s legs are bent at 90° angle from the bed. The extremity on the paretic side will lower earlier.
- This is also useful to test for psychogenic neurologic deficits, whereby in organic paresis, the leg falls but contraction of hamstring muscle is seen. However, in functional paresis, leg falls without contraction of the hamstrings or no fall at all.

### Motor Reflexes

Reflexes are divided into deep tendon reflex (DTR), clonus, cutaneous reflex and frontal lobe release phenomena.

#### DTR

The standardise measurement of DTR is based on the National Institute of Neurological Disorders and Stroke (NINDS) (36, 37) (Table 4). The assessments include jaw jerk, upper limbs and lower limbs.

#### Jaw jerk (first described by Morris J Lewis (38), and reiterated by Lanska (39))

- Place an index finger or thumb over the middle of patient’s chin, holding the mouth open about midway with the jaw relaxed, then tap the finger with the reflex hammer
- The response is a brisk, partial, upward jerk of the mandible caused by contraction of the temporalis, masseter and medial pterygoid muscles (40)

#### Upper limbs

##### Biceps reflex (41, 42)

- Place a slight tension on the patient’s biceps tendon and strike with the reflex hammer.

##### Brachioradialis reflex (41, 42)

- Cradle the patient’s forearm in one hand
- Tap just above the styloid process of the radius with the forearm in semiflexion and semipronation

##### Triceps reflex (41, 42)

- The patient’s wrist has to be across the chest with the elbow about 90° flexion
- Tap the triceps brachial tendon directly above the olecranon process

##### Finger flexor reflex (Wartenberg sign) (42, 43)

- The patient’s hand is in supination, resting on a solid surface, with the fingers slightly flexed
- The examiner places his or her fingers against the patient’s fingers, and taps the back of the examiner’s own fingers lightly

##### Hoffman sign (23, 44)

- The patient’s hand is held with the wrist dorsiflexed and fingers dorsiflexed
- With another hand, the examiner holds the partially extended middle finger of the patient between his or her index finger and thumb. Then, snaps the nail of patient’s middle finger
- Observe for flexion of all fingers, including the thumb in a pathological response

##### Trömner sign (23, 45)

- The examiner holds the partially extended middle finger of the patient, letting the hand dangle
- Then, with the other hand, thumps or flicks the finger pad upward
- Observe for flexion of all fingers, including the thumb in a pathological response

##### Juster sign (as published by Kolář et al. (46))

- The examiner holds the patient’s hand in supination with the fingers fully extended. Then with the other hand, the examiner moves a sharp object over the palm from the wrist across the hypothenar eminence in a direction below the fingers and above the metacarpal heads
- Observe for adduction and opposition of an extended thumb in a pathological response

#### Lower limbs (first described by Erb (47) and Westphal (48))

##### Patellar (quadriceps, knee jerk) reflex (reiterated by Gower (49, 50))

- If the patient is supine, this reflex is easier to elicit with the patient’s knee in slight flexion. A knee jerk can be enhanced by slipping the examiner’s hand under the patient’s knee and resting it on the patient’s opposite thigh
- The examiner then strikes the patella tendon just below the patella of the patient’s relaxed leg
- Observe for contraction of the quadriceps femoris muscle

##### Achilles (ankle jerk) reflex

- The patient is either in a supine or seated position, the thigh should be held in moderate abduction and at external rotation, and the knee is flexed
- Strike the Achilles tendon just above its insertion on the calcaneus

#### Clonus (first described by Erb (47) and Westphal (48))

Clonus is series of rhythmic involuntary muscular contractions induced by the sudden passive stretching of a muscle or tendon (23). The most commonly elicited are at the ankle, patellar and wrist.

##### Ankle

- Position the patient with the knee flexed and the hip externally rotated. The foot should be slightly everted. Then, quickly dorsiflexes the foot (stretch the muscle spindle) while maintaining slight pressure on the sole at the end of the movement
- In most people with normal tone, the foot will not move. If the foot oscillates between flexion and extension for as long as you maintain pressure, then the patient has the sustained clonus (abnormal)

##### Patellar

- Grasps the patient’s patella between the index finger and thumb. Then executes a sudden sharp, downward thrust, holding downward pressure at the end of movement
- The clonus is observed with series of rhythmic up-and-down movement of the patella

##### Fingers or wrist (as published by Szumski et al. (51))

- May be produced by a sudden passive extension of the wrist or fingers

#### Cutaneous reflex (first described by Rosenbach (52), and reiterated by Wartenberg (42, 53))

Cutaneous reflexes response to stimulation of either the skin or mucous membrane. This is a polysynaptic reflex that is performed by a superficial skin stimulus, causing pulling of the umbilicus to the stimulated quadrant. The response is slower and is not consistently present. Dissociation of the abdominal reflexes, with the absence of superficial reflex and exaggerated deep reflexes may suggest a corticospinal tract lesion (23). Absent response may occur in previous abdominal operations, old age, obesity and those who have multiple pregnancies. The most often obtained are superficial abdominal reflex, cutaneous anal reflex and cremasteric reflex.

##### Superficial abdominal reflex

The stimulus is best directed toward the umbilicus. The response is a quick, flicking contraction of the abdominal muscles (rectus abdominis and external oblique) followed by immediate relaxation.

The epigastric region is mediated by intercostal nerves (T5–T7), the supraumbilicalreflexes is mediated by intercostal nerves (T7–T10), while the infraumbilical or suprapubic reflexes by the intercostal, iliohypogastric, and ilioinguinal nerves (T10–L1).

##### Cutaneous anal reflex (anal wink) (S2–S4)

The reflex will cause a contraction of the external sphincter in response to stroking the skin around the anus.

##### Cremasteric reflex (L1–L2) (discovered by Jastrowitz (54))

- The reflex is elicited by gently stroking the skin on the superior and medial aspect of the thigh. This will stimulate the femoral nerve
- The response consists of contraction of the cremasteric muscle with ipsilateral elevation of testicle toward the inguinal canal
- Abnormal response may indicate a spine injury or L1–L2 or testicular torsion

### Frontal Lobe Release Phenomena (55)

These phenomena are generally demonstrable in patients with diffuse dementing illness due to widespread nonlocalised bilateral hemispheric lesions, especially in the frontal lobes. Some causes include extensive infiltrating neoplasm, normal pressure hydrocephalus, closed head injury and toxic-metabolic encephalopathy. Certain test, such as Glabellar tap or Myerson’s sign (56) is only performed on a conscious patient.

#### Snout (orbicularis oris) reflex

Briskly tapping the upper or lower lip with a finger or percussion hammer. A pursing response is obtained if abnormal.

##### Sucking reflex

Elicited by stroking the lip with a finger or a tongue blade. An abnormal response will show a lip pout, sucking, chewing, or swallowing movements.

##### Grasp reflex

- This reflex is normally seen in infants up to 4 months of age, after which this reflex is inhibited by the frontal lobes
- The technique is by stroking the palm of the patient’s hand (between the thumb and index finger) with the examiner’s fingers
- Abnormal response is when the patient grasps the examiner’s hand and is unable to release the grasp. If it is unilateral, it is indicative of involvement of the contralateral frontal lobe, especially in the premotor cortex; bilateral grasp reflexes indicate a more diffuse bifrontal disorder

##### Palmomental reflex (57)

- Stimulation of the thenar eminence in a proximal to distal direction using a sharp object such as the pointed end of a reflex hammer, key, paper clip, or fingernail
- Abnormal response will cause an involuntary contraction of the mentalis muscle of the chin, that is liken to a grimace

##### Groping reflex

- A rhythmic, oscillating groping movements are seen after the withdrawal of the examiner’s fingers from the patient’s grasp
- In a marked response, the examiner’s hand was grasp tightly (forced grasping reflex) due to the inability to relax the muscle in response to contact

### Plantar Response

In the normal individual, stimulation of the skin of the plantar surface of the foot is followed by plantar flexion of the toes. Where there is disease of corticospinal system, there may be dorsiflexion of the toes, with ± fanning of the lateral four toes. The best position is supine, with hips and knees in extension and heels resting on the bed. The response may be reinforced by rotating the patient’s head to the opposite side (23); the result is interpreted as flexor plantar response, extensor plantar response or Babinski sign, or equivocal plantar response. Different methods can be used to elicit extensor plantar response, including Babinski (23, 58, 59), Bing (60), Chaddock (23, 61), Oppenheim (62), Gordon (63, 64), Gonda (65, 66), Schaefer (67) and Marie-Foix retraction sign (68–70).

Another pathological response is called flexion spastic response. In normal individuals, there is a slight dorsiflexion of the toes or no movement at all. However, the pathological response in patients with pyramidal tract disorder causes a quick plantar flexion of the toes at the metatarsophalangeal joints with fan-like positioning (71). The interphalangeal joint is typically in extension. Different methods can be used including the Rossolimo sign (72, 73), Žukovskij-Kornilov sign (as published by Kolář et al. (46)) and Mendel-Bechterew or dorsocubital sign (71, 74) (Table 5).

## Conclusion

It is crucial to identify the focal neurological finding during the examination of a comatose patient. This can help narrow the involved structural lesion and to determine the urgent steps that are needed at the time, in order to prevent further deterioration. Here, we provide a video ( https://youtu.be/2PbquY7XIFg ) of the essential practical guide to the neurological exam of a comatose patient that would assist to determine the aetiology, location and nature of the neurological lesion.

## Figures and Tables

**Table 1:** The FOUR score

Eye response	
4	Eyelids open or opened, tracking or blinking to command
3	Eyelids open but not tracking
2	Eyelids closed but open to loud voice
1	Eyelids closed but open to pain
0	Eyelids remain closed with pain

**Motor response**	

4	Thumbs-up, fist, or peace sign
3	Localizing to pain
2	Flexion response to pain
1	Extension response to pain
0	No response to pain or generalised myoclonus status

**Brainstem reflexes**	

4	Pupil and corneal reflexes present
3	One pupil wide and fixed
2	Pupil or corneal reflexes absent
1	Pupil and corneal reflexes absent
0	Absent pupil, corneal and cough reflex

**Respiration**	

4	Not intubated, regular breathing pattern
3	Not intubated, Cheyne–Stokes breathing pattern
2	Not intubated, irregular breathing
1	Breathes above ventilator rate
0	Breathes at ventilator rate or apnea

Source: Wijdicks et al. (3)

**Table 2:** Glasgow coma scale (GCS) score

Best eye response (4)	
1	No eye opening
2	Eye opening to pain
3	Eye opening to sound
4	Eyes open spontaneously

**Best verbal response** (5)	

1	No verbal response
2	Incomprehensible sounds
3	Inappropriate words
4	Confused-document the questions and answer given by patient
5	Orientated

**Best motor response (6)**	

1	No motor response
2	Abnormal extension to pain - Adduction of arm, internal rotation of shoulder, pronation of forearm, flexion of wrist, decorticate response – The legs are extended and the feet are inverted and plantarflexed
3	Abnormal flexion to pain – Adduction of arm, internal rotation of shoulder, pronation of forearm, flexion of wrist, decorticate response – The legs are extended and the feet are inverted and plantarflexed
4	Flexion/Withdrawal from pain – Flexion of elbow, supination of forearm, flexion of wrist when supra-orbital pressure applied but not above the pressure point
5	Localising pain – Localising to pain with right upper limb only? Or localizing with both arms?-Compare the motor response to noxious stimuli
6	Obeys commands – Document the given instruction e.g. sticking out tongue, fist, peace

Source: Teasdale and Jennett (4)

**Table 3:** The Ashworth scale (25) and the modified Ashworth scale (26)

Scale	Ashworth scale
1	Slight increase in tone giving a catch when the limb is moved in flexion or extension
2	More marked increase in tone but limb easily flexed
3	Considerable increase in tone – passive movement is difficult
4	Limb rigid in flexion or extension

**Scale**	**Modified Ashworth scale**

0	No increase in muscle tone
1	Slight increase in muscle tone, with a catch and release or minimal resistance at the end of the range of motion when an affected part(s) is moved in flexion or extension
1+	Slight increase in muscle tone, manifested as a catch, followed by minimal resistance through the remainder (less than half) of the range of motion
2	A marked increase in muscle tone throughout most of the range of motion, but affected part(s) are still easily moved
3	Considerable increase in muscle tone, passive movement difficult
4	Affected part(s) rigid in flexion or extension

Sources: Ashworth B. (25); Bohannon and Smith (26)

**Table 4:** Measurement of deep tendon reflex (36, 37)

Grade	Description
0	No response
1+	Diminished
2+	Brisk response, Normal
3+	Brisker than average
4+	A tap elicit a repeating reflex (clonus) always abnormal

Source: Hallett M. (36)

**Table 5:** Methods to elicit plantar response

Methods	Maneuver to elicit extensor plantar response
1. Babinski (23, 58, 59)	Move a blunt object over the heel of lateral aspect of the sole, towards the base of great toes
2. Bing (60)	Make multiple light pinpricks on the dorsum of the foot
3. Chaddock (23, 61)	Move a blunt object over the lateral aspect of the foot, drawing from heel towards the small toe
4. Oppenheim (62)	Press your knuckles down anteromedial surface of tibia from the infrapatellar region to the ankle
5. Gordon (63, 64)	Squeeze the calf muscles momentarily
6. Gonda (65, 66)	Pull the fourth toe outward and downward for a brief time and release suddenly
7. Schaefer (67)	Squeeze hard on the Achilles tendon
8. Marie-Foix retraction (68–70)	In patient with pyramidal lesion, pressure on the toes or vigorous plantar flexion at the ankle leads to flexion at the hip and knee and to attempt to dorsiflex the ankle (triple reflex)

	**Maneuver to elicit flexion spastic response**

9. Rossolimo (72, 73)	Using the tendon hammer, tap the ball of the foot or metatarsal head of the toes. Another way is by giving a quick, lifting snap to the tips of the toes
10. Žukovskij-Kornilov (as published by Kolář et al. (46))	Using the tendon hammer, tap at the center of the sole of the foot
11. Mendel-Bechterew or dorsocubital (71, 74)	Using the tendon hammer, tap the outer aspect of the dorsum of the foot in the region of the cuboid bone (os cuboideum) or over the fourth or fifth metatarsal
